# Design, synthesis, ADME prediction and pharmacological evaluation of novel benzimidazole-1,2,3-triazole-sulfonamide hybrids as antimicrobial and antiproliferative agents

**DOI:** 10.1186/s13065-018-0479-1

**Published:** 2018-11-01

**Authors:** Fawzia Faleh Al-blewi, Meshal A. Almehmadi, Mohamed Reda Aouad, Sanaa K. Bardaweel, Pramod K. Sahu, Mouslim Messali, Nadjet Rezki, El Sayed H. El Ashry

**Affiliations:** 1 Department of Chemistry, Faculty of Science, Taibah University, Medina, 30002 Saudi Arabia; 2 Department of Chemistry, Faculty of Sciences, University of Sciences and Technology Mohamed Boudiaf, Laboratoire de Chimie Et Electrochimie des Complexes Metalliques (LCECM) USTO‑MB, P.O. Box 1505, 31000 El M‘nouar, Oran Algeria; 3 Department of Pharmaceutical Sciences, Faculty of Pharmacy, University of Jordan, Amman, 11942 Jordan; 4 School of Study in Chemistry, Jiwaji University, Gwalior, Madhya Pradesh 474011 India; 5 Department of Chemistry, Faculty of Science, Alexandria University, Alexandria, 21500 Egypt

**Keywords:** 1,2,3-Triazoles, Sulfonamides, Benzimidazoles, Click synthesis, Antimicrobial activity, Antiproliferative activity, ADMET

## Abstract

**Background:**

Nitrogen heterocyclic rings and sulfonamides have attracted attention of several researchers.

**Results:**

A series of regioselective imidazole-based mono- and bis-1,4-disubstituted-1,2,3-triazole-sulfonamide conjugates **4a**–**f** and **6a**–**f** were designed and synthesized. The first step in the synthesis was a regioselective propargylation in the presence of the appropriate basic catalyst (Et_3_N and/or K_2_CO_3_) to afford the corresponding mono-**2** and bis-propargylated imidazoles **5**. Second, the ligation of the terminal C≡C bond of mono-**2** and/or bis alkynes **5** to the azide building blocks of sulfa drugs **3a**–**f** using optimized conditions for a Huisgen copper (I)-catalysed 1,3-dipolar cycloaddition reaction yielded targeted 1,2,3-triazole hybrids **4a**–**f** and **6a**–**f**. The newly synthesized compounds were screened for their in vitro antimicrobial and antiproliferative activities. Among the synthesized compounds, compound **6a** emerged as the most potent antimicrobial agent with MIC values ranging between 32 and 64 µg/mL. All synthesized molecules were evaluated against three aggressive human cancer cell lines, PC-3, HepG2, and HEK293, and revealed sufficient antiproliferative activities with IC_50_ values in the micromolar range (55–106 μM). Furthermore, we conducted a receptor-based electrostatic analysis of their electronic, steric and hydrophobic properties, and the results were in good agreement with the experimental results. In silico  ADMET prediction studies also supported the experimental biological results and indicated that all compounds are nonmutagenic and noncarcinogenic.

**Conclusion:**

In summary, we have successfully synthesized novel targeted benzimidazole-1,2,3-triazole-sulfonamide hybrids through 1,3-dipolar cycloaddition reactions between the mono- or bis-alkynes based on imidazole and the appropriate sulfonamide azide under the optimized Cu(I) click conditions. The structures of newly synthesized sulfonamide hybrids were confirmed by means of spectroscopic analysis. All newly synthesized compounds were evaluated for their antimicrobial and antiproliferative activities. Our results showed that the benzimidazole-1,2,3-triazole-sulfonamide hybrids inhibited microbial and fungal strains within MIC values from 32 to 64 μg/mL. The antiproliferative evaluation of the synthesized compounds showed sufficient antiproliferative activities with IC_50_ values in the micromolar range (55–106 μM). In conclusion, compound **6a** has remarkable antimicrobial activity. Pharmacophore elucidation of the compounds was performed based on in silico ADMET evaluation of the tested compounds. Screening results of drug-likeness rules showed that all compounds follow the accepted rules, meet the criteria of drug-likeness and follow Lipinski’s rule of five. In addition, the toxicity results showed that all compounds are nonmutagenic and noncarcinogenic.
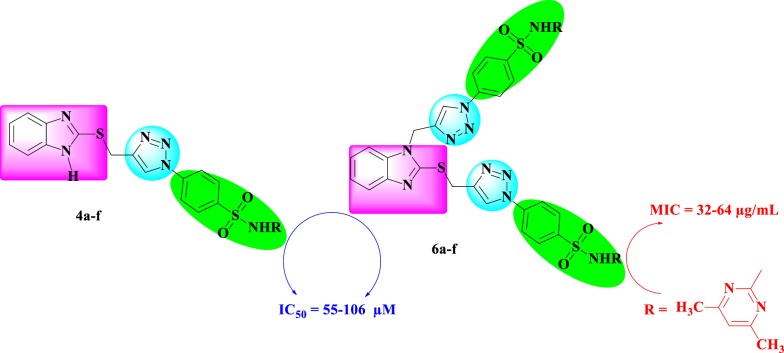

## Background

Currently, a steady increase in the incidences of infectious diseases has occurred due to increasing drug resistance in microbial strains, which has become a major global public health issue [1]. This problem has challenged researchers to develop new antimicrobial agents that will be more potent, more selective and less toxic for combating drug-resistant pathogens. Thus, nitrogen-containing heterocycles, in particular 1,2,3-triazoles [2], have attracted a great deal of interest from medicinal chemists in the design of potential drug candidates owing to their high biocompatibility and various pharmacological actions such as antibacterial [3], antiviral [4], antifungal [5], antimalarial [6], anti-HIV [7], antiallergic [8], antitubercular [9], CNS depressant [10], analgesic [11], anticonvulsant [12], antihypertensive [13] and antiproliferative activities [14].

In addition, 1,2,3-triazoles, attractive linkers that can tether two pharmacophores to provide innovative bifunctional drugs, have become increasingly useful and important in constructing bioactive and functional compounds [15–20].

On the other hand, benzimidazoles represent an important category of active therapeutic agents because their structures are well-suited for biological systems [21]. Their derivatives show various biological activities including antiviral [22], antifungal [23], antiproliferative [24], antihypertensive [25], analgesic [26], anti-inflammatory [27], antibacterial [28] and anthelmintic activities [29].

**Fig. 1:**
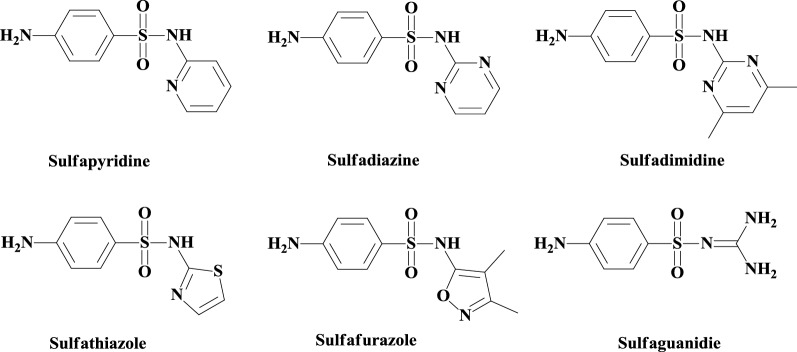
Structure of some sulfa drugs

Sulfonamides, known as sulfa drugs (Fig. 1), are the oldest drugs commonly employed and systematically used as preventive and chemotherapeutic agents against various diseases [30, 31]. Generally, these compounds are easy to prepare, stable and bioavailable, which may explain why such a large number of drugs contain this functionality [32–34].

Among their most important effects, they have been reported to exhibit antiproliferative [35], antibacterial [36], antiviral [37], antiprotozoal [38], antifungal [39], and anti-inflammatory [40] properties. Some important sulfonamide derivatives are also effective for the treatment of urinary diseases, intestinal diseases, rheumatoid arthritis [41], obesity [42] and Alzheimer’s disease [43].

Based on the aforementioned data and as an extension of our studies on the development of novel bioactive 1,2,3-triazoles [44–49], we report herein the design of compounds containing 1,2,3-triazole, benzimidazole and sulfonamides moieties in one scaffold via a Cu(I)-catalysed 1,3-dipolar cycloaddition reaction of sulfa drug azides with propargylated benzimidazoles derivatives and the synergistic effects of the moieties. The newly designed 1,2,3-triazole hybrids have been examined for their antimicrobial and antiproliferative activities.

## Results and discussion

### Chemistry

**Scheme 1:**
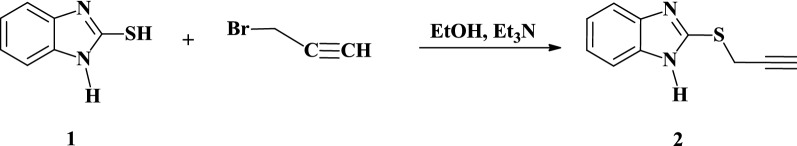
Synthesis of thiopropargylated benzimidazole **2**

**Scheme 2:**
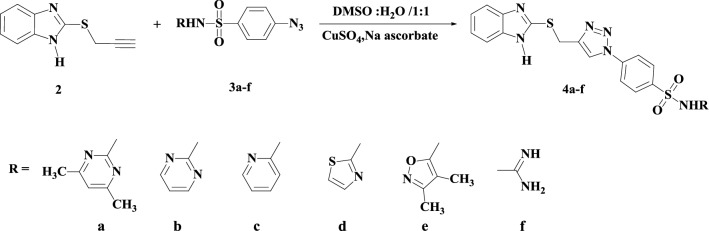
Synthesis of mono-1,4-disubstituted-1,2,3-triazole tethered benzimidazole-sulfonamide conjugates **5a**–**f**

**Scheme 3:**
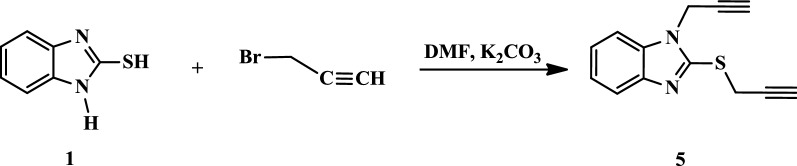
Synthesis of *S,N*-Bispropargylated benzimidazole **5**

**Scheme 4:**
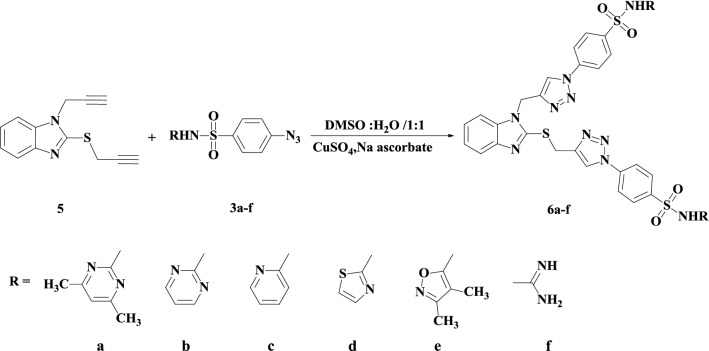
Synthesis of *S,N*-bis(1,2,3-triazole-sulfonamide)-benzimidazole hybrids **6a**–**f**

The target 1,2,3-triazole hybrids (**4a**–**f** and **6a**–**f**) were synthesized by using commercially available 2-mercaptobenzothiazole (**1**) as the starting material as depicted in Schemes 1, 2, 3 and 4. First, the thiol functionality in the 2-position of compound **1** was regioselectively alkylated with propargyl bromide in the presence of triethylamine as a basic catalyst in refluxing ethanol for 1 h to afford target thiopropargylated benzimidazole **2** in 94% yield (Scheme 1). It should be noted that the regioselective synthesis of the thiopropargylated benzimidazole **2** has been previously described using different reaction conditions (NaOH/H_2_O, K_2_CO_3_, H_2_O) [50–52].

The structure of compound **2** was assigned based on its spectral data. The IR spectrum confirmed that **1** had been monopropargylated based on the characteristic NH absorption band at 3390 cm^−1^. The spectrum also revealed the presence of two sharp bands at 3390 and 2140 cm^−1^ related to the acetylenic hydrogen (≡C–H) and the C≡C group, respectively.

The ^1^H NMR analysis clearly confirmed one propargyl side chain had been incorporated at the sulfur atom of **1** based on the presence of one exchangeable proton in the downfield region (δ_H_12.65 ppm) attributable to the triazolyl N**H** proton. The propargyl sp-C**H** and SC**H**_**2**_ protons were assigned to the two singlets at δ_H_ 3.20 and 4.16 ppm, respectively. The four benzimidazole protons were observed at their appropriate chemical shifts (7.14–7.51 ppm). The ^13^C NMR analysis confirmed the incorporation of a propargyl residue by the appearance of diagnostic carbon signals at δ_C_ 20.6, 74.5 and 80.5 ppm, which were attributed to the alkyne S**C**H_2_ and **C**≡**C** groups, respectively. The signals observed at δ_C_ 110.9–148.8 ppm were associated with aromatic and **C**=N carbons.

An azide–alkyne Huisgen cycloaddition reaction was carried out by simultaneously mixing thiopropargylated benzimidazole **2** with the appropriate sulfa drug azide (**4a**–**f**), copper sulfate and sodium ascorbate in DMSO/H_2_O to regioselectively furnish target mono-1,4-disubstituted-1,2,3-triazole tethered benzimidazole-sulfonamide conjugates **5a**–**f** in 85–90% yields after 6–8 h of heating at 80 °C (Scheme 2). The sulfonamide azides were prepared via the diazotization of the appropriate sulfa drugs in a sodium nitrite solution in acidic media followed by the addition of sodium azide.

The formation of compounds **4a**–**f** was confirmed based on their spectroscopic data (IR, ^1^H NMR and ^13^C NMR). Their IR spectra revealed the disappearance of peaks belonging to C≡C at 2140 cm^−1^ and ≡C–H at 3310 cm^−1^, confirming their involvement in the cycloaddition reaction.

The ^1^H NMR spectra of compounds **4a**–**f** revealed the disappearance of the signal attributed to the ≡C–H proton at δ_H_ 3.20 ppm of the precursor *S*-alkyne **2** and the appearance of one singlet at δ_H_ 8.81–8.87 ppm, which was assigned to the 1,2,3-triazole C**H** proton. Instead of a signal for the triazolyl C**H**-proton, the spectra showed two singlets at δ_H_ 4.70–4.81 and 10.85–12.08 ppm due to the SC**H**_**2**_ protons and the imidazolic N**H** proton, respectively. Additionally, the signals of two sp-carbons at 74.5 and 80.5 ppm and the S**C**H_2_-carbon at 20.2–26.3 ppm had disappeared from the ^13^C NMR spectra. New signals were also observed in the aromatic region, and they were assigned to the sp^2^ carbons of the sulfa drug moiety.

The strategy for synthesizing target *S,N*-bis-1,2,3-triazoles **6a**–**f** was based on the regioselective alkylation of **1** with two equivalents of propargyl bromide in the presence of two equivalents of potassium carbonate as a basic catalyst according to our reported procedure [53]. Thus, propargylation of compound **5** by propargyl bromide in the presence of K_2_CO_3_ in DMF afford *S,N*-bispropargylated benzimidazole **5** in 91% yield after stirring at room temperature overnight (Scheme 3).

The absence of the SH and NH stretching bands in the IR spectrum of compound **5**, and the appearance of the characteristic C≡C and ≡C–H bands at 2150 and 3320 cm^−1^, respectively, confirmed the incorporation of two alkyne side chains.

In the ^1^H NMR spectrum of compound **5**, the absence of the S**H** and N**H** protons confirmed the success of the bis-alkylation reaction. The terminal hydrogens of the two ≡C–**H** groups appeared as singlets at δ_H_ 2.29 and 2.40 ppm. The thiomethylene protons (–SC**H**_**2**_) resonated as a distinct upfield singlet at δ_H_ 4.14 ppm. The ^1^H NMR spectrum also revealed the presence of a singlet at δ_H_ 4.93 ppm that integrated to two protons attributable to the NC**H**_**2**_ group. In the ^13^C NMR spectrum of compound **5**, the signals characteristic of the sp **C**≡**C** carbons resonated at δ_C_ 72.3–78.5 ppm, while the S**C**H_2_ and N**C**H_2_ carbons appeared at δ_C_ 21.8 and 33.6 ppm, respectively. Additional signals were also observed in the aromatic region (δ_C_ 109.3–149.1 ppm), and these were attributed to the carbons in the benzimidazole ring.

The *S,N*-bis(1,2,3-triazole-sulfonamide)-benzimidazole hybrids (**6a**–**f**) were synthesized using the same click procedure as described above (Scheme 4). However, the synthesis was conducted using two equivalents of sulfa drug azides **3a**–**f** by a copper-mediated Huisgen 1,3-dipolar cycloaddition reaction in the presence of copper sulfate and sodium ascorbate, and this reaction generated 1,4-disubstituted 1,2,3-triazoles **6a**–**f** in 82–88%.

The structures of *S,N*-bis(1,2,3-triazoles) **6a**–**f** were established on the basis of their spectral data, which indicated the presence of two 1,2,3-triazole moieties based on the absence of the signals for C≡C and ≡C–H at 2150 and 3320 cm^−1^, respectively.

The ^1^H NMR spectra of compounds **6a**–**f** confirmed the presence of the two alkyne linkages between the two 1,2,3-triazole rings based on the disappearance of the sp-carbon signals and the appearance of two triazolyl CH-protons at δ_H_ 8.85–8.93 ppm. The SC**H**_**2**_ and NC**H**_**2**_ protons were assigned to the two singlets at δ_H_ 4.77–4.80 and 5.54–5.58 ppm, respectively. The aromatic protons of the sulfa drug moieties appeared in the appropriate aromatic region. The chemical structures of compounds **6a**–**f** were further elucidated from their ^13^C NMR spectra, which revealed the presence of SC**H**_**2**_ and NC**H**_**2**_ carbon signals at δ_C_ 26.6–27.2 and 40.1–42.3 ppm, respectively. In the cyclization of **5** to **6a**–**f**, the terminal sp carbons disappeared, and new signals that could be assigned to the sulfa drug moieties appeared in the downfield region.

## Biological study

### Antimicrobial screening

An antimicrobial screening against a group of pathogenic microorganisms, including Gram-positive bacteria, Gram-negative bacteria, and fungi, was carried out for the newly synthesized compounds, and the results are summarized in Table 1. Antimicrobial activities are presented as the minimum inhibitory concentrations (MICs), which is the lowest concentration of the examined compound that resulted in more than 80% growth inhibition of the microorganism [54, 55]. In general, the mono-1,2,3-triazole derivatives (**4a**–**f**) exhibited less potent antimicrobial activities than their bis-1,2,3-triazoles (**6a**–**f**) counterparts; this could be attributed to the synergistic effect of the sulfonamoyl and tethered heterocyclic components in addition to the improved lipophilicity of the bis-substituted derivatives.

**Table 1:** Antimicrobial screening results of compounds **4a**–**f** and **6a**–**f** presented as MIC (μg/mL)

Compd. no	Gram-positive organisms		Gram-negative organisms		Fungi organisms	
	*Bc*	*Sa*	*Pa*	*Ec*	*Ab*	*Ca*
**4a**	64	64	256	128	128	128
**4b**	128	128	128	128	256	256
**4c**	256	128	256	64	256	256
**4d**	256	128	256	64	256	256
**4e**	256	128	256	64	256	256
**4f**	512	512	256	256	512	512
**6a**	32	32	64	64	64	32
**6b**	64	64	64	64	128	128
**6c**	128	64	128	32	256	256
**6d**	128	64	128	32	256	256
**6e**	128	64	128	32	256	256
**6f**	256	256	128	128	256	256
Ciprofloxacin	8	4	8	4	–	–
Fluconazole	–	–	–	–	8	4

### Antiproliferative screening

The newly synthesized compounds were examined for their in vitro antiproliferative activity against a human prostate cancer cell line (PC-3), a human liver cancer cell line (HepG2), and a human kidney cancer cell line (HEK293). The correlation between the percentage of proliferating cells and the drug concentration was plotted to generate the proliferation curves of the cancer cell lines. The IC_50_ values were calculated and were defined as the response parameter that corresponds to the concentration required for 50% inhibition of cell proliferation. The results are presented in Table 2.

**Table 2:** In vitro antiproliferative activities (IC_50_ represented as μM ± SD) of the newly synthesized compounds against three human cancer cell lines

Compd. no	IC_50_ PC-3	IC_50_ HepG2	IC_50_ HEK293
**4a**	66	66	70
**4b**	61	62	65
**4c**	80	77	81
**4d**	90	91	92
**4e**	85	86	83
**4f**	104	106	103
**6a**	61	61	64
**6b**	55	56	59
**6c**	73	70	74
**6d**	82	83	84
**6e**	77	79	75
**6f**	95	97	95

IC_50_ values are presented as mean values of three independent experiments. SD were < 10%

Sulfonamides are a valuable chemical scaffold with numerous pharmacological activities including antibacterial, anticarbonic anhydrase, diuretic, hypoglycaemic, and antithyroid activity [56–58]. Notably, structurally novel sulfonamide analogues have been shown to possess significant antitumour activities both in vitro and in vivo*. S*everal mechanisms, such as an anti-angiogenesis effect via matrix metalloproteinase inhibition, carbonic anhydrase inhibition, cell cycle arrest and the disruption of microtubule assembly, have been proposed to explain this interesting activity [59–61].

Interestingly, the newly synthesized compounds exhibited considerable antiproliferative activities against the three cancer cell lines used in this study with IC_50_ values ranging from 55 to 106 μM. Further investigation should shed light on the exact mechanism through which the antiproliferative activity is exerted.

### POM analysis

#### Prediction of pharmacologically relevant inhibition

POM theory is robust and available method to confirm the reliability of experimental data. In actuality, the benefit of POM theory is the ability to predict the biological activities of molecules and easily establish the relationship between steric and electrostatic properties and biological activity. Evaluation of in silico physicochemical properties or ADMET (adsorption, distribution, metabolism, excretion and toxicity) is a robust tool to confirm the potential of a drug candidate [62]. Drug-likenesses of a library of compounds were evaluated by Lipinski’s rule of five, and 90% of orally active compounds follow Lipinski’s rule of five [63]. As per Lipinski’s rule of five, an orally administered drug should have a log *P* ≤ 5, a molecular weight (MW) < 500 Daltons and an HBD  ≤  5 [63] to be in the acceptable range. Results have shown that all compounds have in good agreement in term of HBD, except compound **6a**. This set of criteria is also called Veber’s rule. However, compounds that meet the criteria, i.e., topological polar surface area (TPSA) ≤ 140 Å, are expected to have appropriate oral bioavailability [64]. TPSA is a parameter used to predict the transport properties of drugs in passive molecular transport [64]. The compounds that showed good oral bioavailability or cell permeability were those having TPSA values between 118 and 155 for **4a**–**f** and 197–271 for **6a**–**f** (Table 3).

**Table 3:** In silico prediction of the synthesized sulfonamides **4a**–**f** and **6a**–**f**

Compd. no	MW (g/mol)	Physicochemical properties						Drug likeness					
		TPSA	O/NH	VIOL	VOL	HBA	HBD	GPC	ICM	KI	NRL	PI	EN
**4a**	493	131	2	0	404	10	2	− 0.15	− 0.60	− 0.25	− 0.72	− 0.44	− 0.08
**4b**	465	131	2	0	371	10	2	− 0.10	− 0.45	− 0.13	− 0.73	− 0.40	− 0.00
**4c**	464	118	2	0	375	9	2	− 0.04	− 0.38	− 0.16	− 0.68	− 0.24	0.06
**4d**	470	118	2	0	365	9	2	− 0.27	− 0.59	− 0.21	− 0.86	− 0.37	0.00
**4e**	481	131	2	0	389	12	2	− 0.05	− 0.62	− 0.39	− 0.83	− 0.46	− 0.21
**4f**	428	155	5	0	341	12	5	− 0.04	− 0.22	− 0.16	− 0.82	0.09	0.08
**6a**	834	223	2	2	680	20	8	− 2.04	− 3.48	− 2.85	− 3.16	− 1.60	− 2.52
**6b**	778	223	2	2	615	20	2	− 1.27	− 2.62	− 1.87	− 2.24	− 0.98	− 1.68
**6c**	777	197	2	2	623	18	2	− 1.23	− 2.57	− 1.89	− 2.22	− 0.89	− 1.62
**6d**	789	197	2	2	604	18	2	− 1.06	− 2.29	− 1.53	− 1.91	− 0.73	− 1.30
**6e**	813	223	2	2	652	20	2	− 1.53	− 3.15	− 2.46	− 2.76	− 1.36	− 2.17
**6f**	706	271	8	2	555	20	2	− 0.41	− 1.37	− 0.87	− 1.23	− 0.10	− 0.69
Cipro.	331	75	2	0	285	6	2	− 0.12	− 0.04	− 0.07	− 0.19	− 0.20	0.28
Fluco.	306	82	1	0	249	7	1	− 0.04	− 0.01	− 0.09	− 0.23	− 0.09	0.03

TPSA, total polar surface area; O/NH, O–HN interaction; VIOL, number of violation; VOL, volume; GPC, GPCR ligand; ICM, ion channel modulator; KI, kinase inhibitor; NRL, nuclear receptor ligand; PI, protease inhibitor; EI, enzyme inhibitor; Cipro., Ciprofloxacin; Fluco., Fluconazole; number of hydrogen bond donor (HBD) and acceptor (HBA)

As shown in Table 3, the drug likeness values of the synthesized compounds are larger than that of the standard. The overall drug score (DS) values calculated for sulfonamides **4a**–**f** and **6a**–**f** used ciprofloxacin and fluconazole as the standard drugs, as shown in Table 3. Better drug scores indicate that the compound is more likely to become a drug candidate.

#### In silico bioavailability prediction and cLog*P*

The hydrophilicity and cLog*P* values are correlated because hydrophilicity depends on and is expressed in term of the cLog*P* value. As cLog*P* increases above 5, absorption and permeability decrease. From Table 4, it is clear that our synthesized all sulfa drugs are in the accepting range i.e., lower than 5 (between 0.75 and 4.41) and are potentially active against various biotargets (GPCRL: GPCR ligand; ICM: ion channel modulator; KI: kinase inhibitor; NRL: nuclear receptor ligand; PI: protease inhibitor; and EI: enzyme inhibitor), which confirm the good permeability of all tested molecules. To confirm the reliability of the cLog*P* values and the agreement of these values with the bioavailability, we determined four combine parameters, i.e., the Lipinski, Ghose [65] and Veber rules [66] and the bioavailability score [67], and the results are summarized in Table 4. It is clear from Table 4 that only sulfa drugs **4a**–**f** follow Lipinski rule. Likewise, only sulfa drugs **4a**–**h** follow the Ghose’s rule. In contrast, the screening process showed that none of the sulfa drugs follow Veber’s rule in term of agreement with the in silico bioavailability.

**Table 4:** In silico bioavailability prediction and cLog*P* value

Compd. no	*In silico* Bioavailability and cLog*P*				Bioavailability score
	cLog*P*	Lipinski	Ghose	Veber	
**4a**	3.19	Yes	No	No	0.55
**4b**	2.31	Yes	Yes	No	0.55
**4c**	3.24	Yes	Yes	No	0.55
**4d**	3.19	Yes	Yes	No	0.55
**4e**	3.34	Yes	No; 1 violation	No	0.55
**4f**	1.52	Yes	Yes	No	0.55
**6a**	4.11	No	No	No	0.17
**6b**	2.35	No	No	No	0.17
**6c**	4.20	No	No	No	0.17
**6d**	4.10	No	No	No	0.17
**6e**	4.41	No	No	No	0.17
**6f**	0.75	No	No	No	0.17
Cipro.	− 0.70	Yes	Yes	Yes	0.55
Fluco.	− 0.12	Yes	Yes	Yes	0.55

#### In silico pharmacokinetic analysis of the synthesized sulfonamides

Due to poor pharmacokinetics, most drugs fail to move into clinic trials in the discovery process. Pharmacokinetics determine the human therapeutic use of compounds, and these properties depend on the absorption, distribution, metabolism, excretion, and toxicity (ADMET) properties [68, 69], which is why in silico pharmacokinetic studies are necessary to minimize the possibility of failure of any drug in clinical trials. In silico pharmacokinetic has explained in term of ADME/T and toxicity. Further analyzed in silico data has been correlated and found in good agreement (Table 5).

**Table 5:** In silico pharmacokinetics prediction of sulfonamides

Compd. no	In silico pharmacokinetics					
	GI absorption	BBB permeant	P-gp	CYP1A2 inhibitor	CYP2D6 inhibitor	Log *K*_p_ (skin permeation), cm/s
4a	Low	No	Yes	No	No	− 7.01
**4b**	Low	No	Yes	No	No	− 7.42
**4c**	Low	No	Yes	Yes	No	− 6.95
**4d**	Low	No	No	No	No	− 6.91
**4e**	Low	No	No	No	No	− 6.85
**4f**	Low	No	No	No	No	− 7.81
**6a**	Low	No	Yes	No	No	− 8.43
**6b**	Low	No	Yes	No	No	− 9.23
**6c**	Low	No	Yes	No	No	− 8.29
**6d**	Low	No	Yes	No	No	− 8.22
**6e**	Low	No	Yes	No	No	− 8.11
**6f**	Low	No	No	No	No	− 10.03
Cipro.	High	No	Yes	No	No	− 9.09
Fluco.	High	No	No	No	No	− 7.92

GI, gastro intestinal; P-gp, P-glycoprotein; BBB, blood brain barrier; CYP1A2, cytochrome P450 family 1 subfamily A member 2 (PDB: 2HI4); CYP2D6, cytochrome P450 family 2 subfamily D member 6 (PDB: 5TFT)

#### In silico toxicity analysis

In silico carcinogenicity has been evaluated and tabulated in Table 6. It was found that all the synthesized sulfonamides were noncarcinogenic. In Table 6, the green colour indicates drug-like behaviour. For further investigation of the in vivo antimicrobial activity, the computed LD_50_ in rat from the acute toxicity model seems to be sufficiently safe (2.29–2.41 mol/kg).

**Table 6:** In silico predicted LD_50_ and toxicity profile of the synthesized sulfonamides **4a**–**f** and **6a**–**f** [70]

Compd. no	AMES toxicity	Carcinogenicity	Rat acute toxicity LD_50_, (mol/kg)
**4a**			2.30
**4b**			2.30
**4c**			2.38
**4d**			2.29
**4e**			2.31
**4f**			2.34
**6a**			2.41
**6b**			2.34
**6c**			2.41
**6d**			2.35
**6e**			2.41
**6f**			2.36

## Materials and methods

### General methods

Melting points were measured on a melt-temp apparatus (SMP10) and are uncorrected. TLC analyses were performed on silica gel-coated aluminium plates (Kieselgel, 0.25 mm, 60 F254, Merck, Germany), and spots were visualized by ultraviolet (UV) light absorption using a developing solvent system of ethyl acetate/hexane. The IR spectra were measured in a KBr matrix using a SHIMADZU FTIR-8400S spectrometer. ^1^H NMR spectra were recorded using an Advance Bruker NMR spectrometer at 400–600 MHz, whereas ^13^C NMR spectra were recorded on the same instrument at 100–150 MHz using tetramethylsilane (TMS) as the internal standard. High-resolution mass spectrometry (HRMS) was carried out using an LC–MS/MS impact II.

### Synthesis and characterization of 2-(prop-2-yn-1-ylthio)-1*H*-benzo[*d*]imidazole (**2**)

To a solution of 2-mercaptobenzimidazole (**1**) (10 mmol) in ethanol (40 mL) and triethylamine (Et_3_N) (12 mmol) was added propargyl bromide (12 mmol) with stirring, and the solution was heated to reflux for 1 h. The excess solvent was removed under reduced pressure, and the resulting crude product was washed with water and recrystallized from ethanol to afford compound **2** in 94% yield as colourless crystals, mp: 163–164 °C (lit. 164–165 °C [50, 51]); IR (KBr) υ_max_/cm^−1^ 1580 (C=C), 1615 (C=N), 2140 (C≡C), 2950 (C–H al), 3070 (C–H Ar), 3310 cm^−1^ (≡CH), 3390 cm^−1^ (N–H). ^1^H NMR (400 MHz, DMSO-*d*_6_) δ_H_ = 3.20 (s, 1H, ≡CH), 4.16 (s, 2H, SCH_2_), 7.14–7.16 (m, 2H, Ar–H), 7.46–7.51 (m, 2H, Ar–H), 12.65 (s, 1H, NH). ^13^C NMR (100 MHz, DMSO-*d*_6_) δ_C_ = 20.6 (SCH_2_); 74.5, 80.5 (C≡C); 110.9, 118.0, 122.1, 122.6, 135.9, 144.1, 148.8 (Ar–C, C=N). HRMS (ESI): 188.0410 [M^+^].

### Synthesis of 1,4-disubstituted mono-1,2,3-triazoles 4**a**–**f**

To a solution of compound **2** (1 mmol) in a 1:1 mixture of dimethyl sulfoxide (DMSO) and water (20 mL), CuSO_4_ (0.10 g) were added Na ascorbate (0.15 g) and the appropriate sulfonamide azide (**3a**–**f**, 1 mmol) with stirring. The resulting mixture was stirred at 80 °C for 6–8 h. The consumption of the starting materials was monitored using TLC. The reaction mixture was quenched with water, and the solid thus formed was collected by filtration, washed with a saturated solution of sodium chloride and recrystallized from ethanol to give the desired 1,2,3-triazoles (**4a**–**f**).

*4*-*(4*-*((1H*-*Benzo[d]imidazol*-*2*-*ylthio)methyl)*-*1H*-*1,2,3*-*triazol*-*1*-*yl)*-*N*-*(4,6*-*dimethylpyrimidin*-*2*-*yl)benzenesulfonamide* (***4a***). White solid; Yield: 90%; mp: 153–154 °C; IR (KBr) υ_max_/cm^−1^ 1580 (C=C), 1620 (C=N), 2935 (C–H al), 3045 (C–H Ar), 3340–3385 cm^−1^ (N–H). ^1^H NMR (400 MHz, DMSO-*d*_6_) δ_H_ = 2.26 (s, 6H, 2 × CH_3_), 4.76 (s, 2H, SCH_2_), 6.73 (bs, 1H, Ar–H), 7.13 (bs, 2H, Ar–H), 7.44–7.54 (m, 2H, Ar–H), 7.89–8.13 (m, 4H, Ar–H), 8.86 (bs, 1H, CH-1,2,3-triazole), 12.04 (bs, 1H, NH), 12.86 (s, 1H, NH). ^13^C NMR (100 MHz, DMSO-*d*_6_) δ_C_ = 20.2 (CH_3_), 24.7 (SCH_2_), 110.8, 116.1, 117.4, 120.1, 122.3, 122.5, 123.7, 130.0, 138.9, 139.9, 140.2, 142.8, 143.3, 154.0, 164.2 (Ar–C, C=N). HRMS (ESI): 492.1296 [M^+^].

*4*-*(4*-*((1H*-*Benzo[d]imidazol*-*2*-*ylthio)methyl)*-*1H*-*1,2,3*-*triazol*-*1*-*yl)*-*N*-*(pyrimidin*-*2*-*yl)benzenesulfonamide* (***4b***). White solid; Yield: 87%; mp: 165–166 °C; IR (KBr) υ_max_/cm^−1^ 1585 (C=C), 1625 (C=N), 2910 (C–H al), 3065 (C–H Ar), 3330–3395 cm^−1^ (N–H). ^1^H NMR (400 MHz, DMSO-*d*_6_) δ_H_ = 4.74 (s, 2H, SCH_2_), 7.06–7.13 (m, 3H, Ar–H), 7.50 (bs, 2H, Ar–H), 8.11–8.16 (bs, 4H, Ar–H), 8.52 (bs, 2H, Ar–H), 8.87 (s, 1H, CH-1,2,3-triazole), 12.08 (bs, 1H, NH), 12.69 (bs, 1H, NH). ^13^C NMR (100 MHz, DMSO-*d*_6_) δ_C_ = 26.3 (SCH_2_), 110.6, 116.1, 117.2, 120.6, 122.0, 122.4, 125.9, 129.9, 139.6, 140.1, 140.4, 142.6, 143.5, 155.2, 163.9 (Ar–C, C=N). HRMS (ESI): 464.1272 [M^+^].

*4*-*(4*-*(((1H*-*Benzo[d]imidazol*-*2*-*yl)thio)methyl)*-*1H*-*1,2,3*-*triazol*-*1*-*yl)*-*N*-*(pyridin*-*2*-*yl)benzenesulfonamide* (***4c***). White solid; Yield: 85%; mp: 216–218 °C; IR (KBr) υ_max_/cm^−1^ 1575 (C=C), 1610 (C=N), 2930 (C–H al), 3040 (C–H Ar), 3290–3365 cm^−1^ (N–H). ^1^H NMR (600 MHz, DMSO-*d*_6_) δ_H_ = 4.70 (s, 2H, SCH_2_), 6.84 (bs, 1H, Ar–H), 7.11–7.21 (m, 3H, Ar–H), 7.40–7.54 (m, 2H, Ar–H), 7.75 (m, 1H, *J* = 6 Hz, Ar–H), 7.84–7.95 (m, 2H, Ar–H), 7.95–8.10 (m, 4H, Ar–H), 8.81 (s, 1H, CH-1,2,3-triazole), 12.59 (bs, 1H, NH), 12.63 (bs, 1H, NH). ^13^C NMR (100 MHz, DMSO-*d*_6_) δ_C_ = 25.8 (SCH_2_), 110.4, 117.5, 120.3, 121.2, 121.8, 122.0, 125.6, 128.2, 134.2, 135.5, 138.5, 141.8, 144.8, 149.1, 163.5 (Ar–C, C=N). HRMS (ESI): 463.0975 [M^+^].

*4*-*(4*-*((1H*-*Benzo[d]imidazol*-*2*-*ylthio)methyl)*-*1H*-*1,2,3*-*triazol*-*1*-*yl)*-*N*-*(thiazol*-*2*-*yl)benzenesulfonamide* (***4d***). White solid; Yield: 89%; mp: 148–150 °C; IR (KBr) υ_max_/cm^−1^ 1590 (C=C), 1610 (C=N), 2925 (C–H al), 3055 (C–H Ar), 3315–3380 cm^−1^ (N–H). ^1^H NMR (400 MHz, DMSO-*d*_6_) δ_H_ = 4.72 (s, 2H, SCH_2_), 6.86–7.27 (m, 6H, Ar–H), 7.99 (bs, 4H, Ar–H), 8.86 (s, 1H, CH-1,2,3-triazole), 12.52 (bs, 2H, 2 × NH). ^13^C NMR (100 MHz, DMSO-*d*_6_) δ_C_ = 20.2 (SCH_2_), 110.8, 114.2, 116.1, 117.4, 122.3, 122.5, 123.7, 130.0, 135.4, 138.9, 139.9, 140.2, 142.8, 143.3, 154.5, 161.3 (Ar–C, C=N). HRMS (ESI): 469.0896 [M^+^].

*4*-*(4*-*(((1H*-*Benzo[d]imidazol*-*2*-*yl)thio)methyl)*-*1H*-*1,2,3*-*triazol*-*1*-*yl)*-*N*-*(3,4*-*dimethylisoxazol*-*5*-*yl)benzenesulfonamide* (***4e***). White solid; Yield: 88%; mp: 204–206 °C; IR (KBr) υ_max_/cm^−1^ 1570 (C=C), 1620 (C=N), 2975 (C–H al), 3080 (C–H Ar), 3300–3395 cm^−1^ (N–H). ^1^H NMR (600 MHz, DMSO-*d*_6_) δ_H_ = 2.21 (s, 3H, CH_3_), 2.55 (s, 3H, CH_3_), 4.81 (s, 2H, SCH_2_), 7.09–7.16 (m, 2H, Ar–H), 7.49–7.54 (m, 2H, Ar–H), 7.77–7.84 (m, 2H, Ar–H), 7.98–8.03 (m, 2H, Ar–H), 8.87 (s, 1H, CH-1,2,3-triazole), 10.85 (bs, 1H, NH), 13.36 (bs, 1H, NH). ^13^C NMR (150 MHz, DMSO-*d*_6_) δ_C_ = 21.0 (CH_3_), 23.2 CH_3_), 26.3 (SCH_2_), 111.0, 114.0, 117.5, 119.7, 120.5, 122.5, 127.0, 129.5, 135.8, 138.5, 139.7, 140.8, 143.1, 148.9, 162.5 (Ar–C, C=N). HRMS (ESI): 481.0934 [M^+^].

*4*-*(4*-*(((1H*-*Benzo[d]imidazol*-*2*-*yl)thio)methyl)*-*1H*-*1,2,3*-*triazol*-*1*-*yl)*-*N*-*(diaminomethylene)benzenesulfonamide* (***4f***). White solid; Yield: 90%; mp: 244–246 °C;IR (KBr) υ_max_/cm^−1^ 1570 (C=C), 1615 (C=N), 2980 (C–H al), 3025 (C–H Ar), 3265–3380 cm^−1^ (N–H). ^1^H NMR (600 MHz, DMSO-*d*_6_) δ_H_ = 4.73 (s, 2H, SCH_2_), 6.70 (bs, 4H, 2 × NH_2_), 7.13 (dd, 2H, *J* = 6, 12 Hz, Ar–H), 7.48 (bs, 2H, Ar–H), 7.92–8.01 (m, 4H, Ar–H), 8.81 (s, 1H, CH-1,2,3-triazole), 12.57 (bs, 2H, 2 × NH). ^13^C NMR (150 MHz, DMSO-*d*_6_) δ_C_ = 25.9 (SCH_2_), 120.2, 121.60, 122.0, 127.4, 135.7, 138.1, 144.3, 144.8, 148.8, 158.2 (Ar–C, C=N). HRMS (ESI): 428.0841 [M^+^].

### Synthesis and characterization of 1-(prop-2-yn-1-yl)-2-(prop-2-yn-1-ylthio)-1*H*-benzo[*d*]imidazole (5)

A mixture of 2-mercaptobenzimidazole (**1**) (10 mmol), dimethylformamide (DMF) (20 mL) and potassium carbonate (22 mmol) were stirred at room temperature for 2 h. Then, propargyl bromide (24 mmol) was added, and the mixture was stirred overnight at room temperature. The consumption of the starting materials was monitored using TLC. The reaction mixture was poured into crushed ice. The product was collected by filtration, washed with water and recrystallized from ethanol to afford compound **5** in 91% yield as colourless crystals. mp: 72–73 °C (lit. 70–71 °C [53]); 1585 (C=C), 1610 (C=N), 2150 (C≡C), 2930 (C–H al), 3045 (C–H Ar), 3320 cm^−1^ (≡CH). ^1^H NMR (400 MHz, DMSO-*d*_6_) δ_H_ = 2.29 (s, 1H, ≡CH), 2.40 (s, 1H, ≡CH), 4.14 (s, 2H, SCH_2_), 4.93 (s, 2H, NCH_2_), 7.27–7.31 (m, 2H, Ar–H), 7.42–7.45 (m, 1H, Ar–H), 7.73–7.77 (m, 1H, Ar–H). ^13^C NMR (100 MHz, DMSO-*d*_6_) δ_C_ = 21.8 (SCH_2_); 33.6 (NCH_2_); 72.3, 73.8, 76.3, 78.5 (C≡C); 109.3, 118.9, 122.5, 122.7, 135.5, 143.4, 149.1 (Ar–C, C=N). HRMS (ESI): 226.0569 [M^+^].

### Synthesis of 1,4-disubstituted bis-1,2,3-triazoles 6**a**–**f**

To a solution of compound **5** (1 mmol) in a 1:1 mixture of dimethyl sulfoxide (DMSO) and water (20 mL) were added CuSO_4_ (0.20 g), Na ascorbate (0.30 g) and sulfonamide azide (**3a**–**f**, 2 mmol) with stirring. The resulting mixture was stirred at 80 °C for 8–12 h. The consumption of the starting materials was monitored using TLC. The reaction mixture was quenched with water, and the solid thus formed was collected by filtration, washed with a saturated solution of sodium chloride and recrystallized from ethanol to give the desired 1,2,3-triazoles (**6a**–**f**).

*N*-*(4,6*-*Dimethylpyrimidin*-*2*-*yl)*-*4*-*(4*-*((1*-*((1*-*(4*-*(N*-*(4,6*-*dimethylpyrimidin*-*2*-*yl)sulfamoyl)*-*phenyl)*-*1H*-*1,2,3*-*triazol*-*4*-*yl)methyl)*-*1H*-*benzo[d]*-*imidazol*-*2*-*ylthio)methyl)*-*1H*-*1,2,3*-*triazol*-*1*-*yl)benzenesulfonamide* (***6a***). White solid; Yield: 87%; mp: 176–178 °C; IR (KBr) υ_max_/cm^−1^ 1595 (C=C), 1630 (C=N), 2915 (C–H al), 3070 (C–H Ar), 3310–3370 cm^−1^ (N–H). ^1^H NMR (400 MHz, DMSO-*d*_6_) δ_H_ = 2.55 (s, 6H, 2 x CH_3_), 4.78 (s, 2H, SCH_2_), 5.56 (s, 2H, NCH_2_), 6.72 (bs, 2H, Ar–H), 7.20 (bs, 2H, Ar–H), 7.64–7.66 (m, 2H, Ar–H), 8.06–8.15 (m, 8H, Ar–H), 8.87 (s, 1H, CH-1,2,3-triazole), 8.95 (s, 1H, CH-1,2,3-triazole), 12.21 (s, 2H, NH). ^13^C NMR (100 MHz, DMSO-*d*_6_) δ_C_ = 27.2 (SCH_2_), 40.2 (NCH_2_), 23.0 (CH_3_), 110.5, 116.3, 117.5, 120.1, 122.3, 122.4, 122.5, 122.7, 130.2, 135.3, 139.0, 140.3, 142.6, 143.9, 149.4, 154.2, 156.2, 164.6 (Ar–C, C=N). HRMS (ESI): 834.2319 [M^+^].

*N*-*(Pyrimidin*-*2*-*yl)*-*4*-*(4*-*((1*-*((1*-*(4*-*(N*-*pyrimidin*-*2*-*ylsulfamoyl)phenyl)*-*1H*-*1,2,3*-*triazol*-*4*-*yl)*-*methyl)*-*1H*-*benzo[d]imidazol*-*2*-*ylthio)methyl)*-*1H*-*1,2,3*-*triazol*-*1*-*yl)benzenesulfonamide* (***6b***). White solid; Yield: 83%; mp: 199–201 °C; IR (KBr) υ_max_/cm^−1^ 1580 (C=C), 1610 (C=N), 2925 (C–H al), 3040 (C–H Ar), 3320–3375 cm^−1^ (N–H). ^1^H NMR (400 MHz, DMSO-*d*_6_) δ_H_ = 4.78 (s, 2H, SCH_2_), 5.57 (s, 2H, NCH_2_), 7.06 (s, 2H, Ar–H), 7.20 (bs, 2H, Ar–H), 7.63 (bs, 2H, Ar–H), 8.07–8.17 (m, 8H, Ar–H), 8.51 (bs, 4H, Ar–H), 8.88 (s, 2H, 2 × CH-1,2,3-triazole), 12.11 (s, 2H, 2 × NH). ^13^C NMR (100 MHz, DMSO-*d*_6_) δ_C_ = 26.6 (SCH_2_), 40.4 (NCH_2_), 109.8, 116.5, 118.0, 120.1, 122.0, 122.2, 122.6, 123.1, 129.4, 130.4, 135.6, 139.1, 140.2, 142.8, 143.4, 144.5, 149.8, 154.1, 156.6, 165.0 (Ar–C, C=N). HRMS (ESI): 778.12077 [M^+^].

*N*-*(Pyridin*-*2*-*yl)*-*4*-*(4*-*(((1*-*((1*-*(4*-*(N*-*(pyridin*-*2*-*yl)sulfamoyl)phenyl)*-*1H*-*1,2,3*-*triazol*-*4*-*yl)*-*methyl)*-*1H*-*benzo[d]imidazol*-*2*-*yl)thio)methyl)*-*1H*-*1,2,3*-*triazol*-*1*-*yl)benzenesulfonamide* (***6c***). White solid; Yield: 82%; mp: 220–222 °C; IR (KBr) υ_max_/cm^−1^ 1580 (C=C), 1630 (C=N), 2985 (C–H al), 3025 (C–H Ar), 3280–3350 cm^−1^ (N–H). ^1^H NMR (600 MHz, DMSO-*d*_6_) δ_H_ = 4.77 (s, 2H, SCH_2_), 5.54 (s, 2H, NCH_2_), 6.85 (bs, 2H, Ar–H), 7.19–7.26 (m, 4H, Ar–H), 7.61–7.64 (m, 2H, Ar–H), 7.75–7.77 (m, 2H, Ar–H), 7.88–7.92 (m, 2H, Ar–H), 7.97–8.04 (m, 8H, Ar–H), 8.84 (s, 1H, CH-1,2,3-triazole), 8.93 (s, 1H, CH-1,2,3-triazole), 12.41 (s, 2H, NH). ^13^C NMR (150 MHz, DMSO-*d*_6_) δ_C_ = 26.7 (SCH_2_), 40.4 (NCH_2_), 110.1, 117.9, 119.5, 120.3, 120.3, 121.8, 122.0, 122.1, 122.2, 128.2, 128.5, 135.9, 138.4, 142.9, 144.4, 150.3, 154.8, 156.4, 164.7 (Ar–C, C=N). HRMS (ESI): 776.2614 [M^+^].

*N*-*(Thiazol*-*2*-*yl)*-*4*-*(4*-*((1*-*((1*-*(4*-*(N*-*thiazol*-*2*-*ylsulfamoyl)phenyl)*-*1H*-*1,2,3*-*triazol*-*4*-*yl)methyl)*-*1H*-*benzo[d]imidazol*-*2*-*ylthio)methyl)*-*1H*-*1,2,3*-*triazol*-*1*-*yl)benzenesulfonamide* (***6d***). White solid; Yield: 85%; mp: 158–160 °C; IR (KBr) υ_max_/cm^−1^ 1580 (C=C), 1625 (C=N), 2945 (C–H al), 3030 (C–H Ar), 3325–3370 cm^−1^ (N–H). ^1^H NMR (400 MHz, DMSO-*d*_6_) δ_H_ = 4.78 (s, 2H, SCH_2_), 5.56 (s, 2H, NCH_2_), 6.87 (bs, 2H, Ar–H), 7.20–7.29 (m, 4H, Ar–H), 7.61–7.65 (m, 2H, Ar–H), 7.80–8.05 (m, 8H, Ar–H), 8.86 (s, 1H, CH-1,2,3-triazole), 8.95 (s, 1H, CH-1,2,3-triazole), 12.86 (s, 2H, 2 × NH). ^13^C NMR (100 MHz, DMSO-*d*_6_) δ_C_ = 27.2 (SCH_2_), 41.1 (NCH_2_), 109.0, 110.5, 118.3, 119.9, 120.8, 120.9, 122.3, 122.4, 122.5, 122.6, 125.1, 128.0, 128.2, 139.0, 139.1, 142.5, 142.6, 143.4, 143.9, 144.9, 150.8, 154.3, 169.5 (Ar–C, C=N). HRMS (ESI): 788.0685 [M^+^].

*N*-*(3,4*-*Dimethylisoxazol*-*5*-*yl)*-*4*-*(4*-*(((1*-*((1*-*(4*-*(N*-*(3,4*-*dimethylisoxazol*-*5*-*yl)sulfamoyl)phenyl)*-*1H*-*1,2,3*-*triazol*-*4*-*yl)methyl)*-*1H*-*benzo[d]imidazol*-*2*-*yl)thio)*-*methyl)*-*1H*-*1,2,3*-*triazol*-*1*-*yl)benzenesulfonamide* (***6e***). White solid; Yield: 85%; mp: 238–240 °C; IR (KBr) υ_max_/cm^−1^ 1580 (C=C), 1610 (C=N), 2955 (C–H al), 3045 (C–H Ar), 3315–3370 cm^−1^ (N–H). ^1^H NMR (600 MHz, DMSO-*d*_6_) δ_H_ = 2.08 (s, 6H, 2 × CH_3_), 2.57 (s, 3H, CH_3_), 4.80 (s, 2H, SCH_2_), 5.58 (s, 2H, NCH_2_), 7.21–7.23 (m, 2H, Ar–H), 7.53–7.63 (m, 4H, Ar–H), 7.95–8.14 (m, 6H, Ar–H), 8.92 (bs, 2H, 2 × CH-1,2,3-triazole), 10.75 (bs, 1H, NH), 11.17 (bs, 1H, NH). ^13^C NMR (150 MHz, DMSO-*d*_6_) δ_C_ = 18.5 (CH_3_), 21.0 (CH_3_), 26.7 (SCH_2_), 42.3 (NCH_2_), 109.8, 110.1, 117.9, 120.6, 121.9, 122.0, 122.7, 122.8, 128.6, 129.6, 135.9, 139.6, 142.8, 143.6, 144.2, 150.3, 155.0, 168.8 (Ar–C, C=N). HRMS (ESI): 812.1731 [M^+^].

*N*-*(Diaminomethylene)*-*4*-*(4*-*(((1*-*((1*-*(4*-*(N*-*(diaminomethylene)sulfamoyl)phenyl)*-*1H*-*1,2,3*-*triazol*-*4*-*yl)methyl)*-*1H*-*benzo[d]imidazol*-*2*-*yl)thio)methyl)*-*1H*-*1,2,3*-*triazol*-*1*-*yl)benzenesulfonamide* (***6f***). White solid; Yield: 88%; mp: 276–278 °C; IR (KBr) υ_max_/cm^−1^ 1575 (C=C), 1620 (C=N), 2950 (C–H al), 3040 (C–H Ar), 3260–3350 cm^−1^ (N–H). ^1^H NMR (600 MHz, DMSO-*d*_6_) δ_H_ = 4.79 (s, 2H, SCH_2_), 5.58 (s, 2H, NCH_2_), 6.80 (bs, 2H, 2 × NH_2_), 7.19–7.20 (m, 2H, Ar–H), 7.63–7.67 (m, 2H, Ar–H), 7.91–7.98 (m, 8H, Ar–H), 8.85 (s, 1H, CH-1,2,3-triazole), 8.92 (s, 1H, CH-1,2,3-triazole), 12.40 (bs, 2H, NH). ^13^C NMR (150 MHz, DMSO-*d*_6_) δ_C_ = 26.7 (SCH_2_), 40.1 (NCH_2_), 110.1, 111.3, 117.8, 120.1, 120.2, 122.1, 122.5, 122.8, 127.2, 128.3, 135.4, 137.9, 143.2, 144.3, 149.3, 158.0 (Ar–C, C=N). HRMS (ESI): 706.1343 [M^+^].

## Biological activity

### Antimicrobial activity

#### Minimal inhibitory concentration (MIC) determination

The microdilution susceptibility tests were carried out in Müller–Hinton broth (Oxoid) and Sabouraud liquid medium (Oxoid) for the assessment of antibacterial and antifungal activity, respectively. The newly synthesized compounds were evaluated for their antimicrobial activity against four pathogenic bacterial strains [Gram-positive: *Bacillus cereus* (ATTC 10876) and *Staphylococcus aureus* (ATTC 25923) and Gram-negative: *Escherichia coli* (ATTC 25922) and *Pseudomonas aeruginosa* (ATTC 27853)] and two fungal strains [(*Candida albicans* (ATTC 50193) and *Aspergillus brasiliensis* (ATTC 16404)].

The examined compounds were dissolved in DMSO, and stock solutions were prepared at a concentration of 10 mM before being further diluted to the desired concentrations. Ciprofloxacin and fluconazole were used as standard antimicrobial agents. A 10 µL aliquot of the medium containing approximately 10^6^ CFU/mL of each bacterial species or 10^4^ CFU/mL of the test fungus was added to each well of a 96-well microtiter plate. The wrapped microplates were incubated at 37 °C for 24 h to measure antibacterial activity and at 25 °C for 48 h for antifungal activity in a humidified atmosphere. Optical densities were measured at 600 nm (OD_600_) using a microplate reader (Palo Alto, CA, USA). The minimal inhibitory concentrations (MICs) were determined at the end of the incubation period and were defined as the lowest concentration at which more than 80% of the microbial growth was inhibited. MIC assessments were carried out in triplicate and repeated three times for each microorganism, and the SD values never exceeded 5%. Control experiments with the standard antimicrobial agents (positive control) and the uninoculated media (negative control) were performed parallel to the examined compounds and under the same conditions.

### Antiproliferative activity

#### MTT assay

Logarithmically growing cells were washed with PBS, detached from the surface with trypsin, and then transferred to fresh cultured medium containing 10% FBS, 100 U/mL penicillin and 0.1 mg/mL streptomycin. Cells were plated at a density of 1 × 10^4^ cell/well into 96-well culture plates and incubated at 37 °C in an atmosphere containing 5% CO_2_ for 24 h to allow for adhesion. Stock solutions (1 mM) of the newly synthesized compounds were freshly prepared in dimethyl sulfoxide (DMSO) prior to the experiment and applied on the cells at concentrations ranging from 1 to 300 µM. The highest DMSO concentration in the medium (0.1%) did not have any significant effect on cell proliferation. Cells were incubated with the tested compounds for 48 h. Control wells were treated with 0.1% DMSO in medium or the standard antiproliferative agent doxorubicin. At the end of the exposure time (48 h), the medium was removed, and the wells were washed with 200 µL of PBS. Afterward, 100 µL of freshly prepared MTT [(3-[4,5-dimethylthiazole-2-yl]-2,5-diphenyl-tetrazolium bromide)] reagent was added to each well, and the plates were incubated at 37 °C for 4 h. Then, the supernatant was aspirated, and 100% DMSO was added to solubilize the formed formazan crystals. The optical density (OD) was obtained by reading the absorbance using an ELISA plate reader at 540 nm and 670 nm. Cell proliferation percentages were plotted against the examined compound concentrations to determine the IC_50_ values. The human prostate cancer cell line (PC-3), the human liver cancer cell line (HepG2), and the human kidney cancer cell line (HEK293) were used in this study. Each concentration of the examined compounds was tested in triplicate, and IC_50_ values, i.e., concentration of the compound at which 50% inhibition of cell proliferation occurred, was calculated based on the mean value of triplicate readings.

## Conclusions

In summary, we have successfully synthesized novel targeted benzimidazole-1,2,3-triazole-sulfonamide hybrids through 1,3-dipolar cycloaddition reactions between the mono- or bis-alkynes based on imidazole and the appropriate sulfonamide azide under the optimized Cu(I) click conditions. The structures of newly synthesized sulfonamide hybrids were confirmed by means of spectroscopic analysis. All newly synthesized compounds were evaluated for their antimicrobial and antiproliferative activities. Our results showed that the benzimidazole-1,2,3-triazole-sulfonamide hybrids inhibited microbial and fungal strains within MIC values from 32 to 64 μg/mL. The antiproliferative evaluation of the synthesized compounds showed sufficient antiproliferative activities with IC_50_ values in the micromolar range (55–106 μM). In conclusion, compound 6a has remarkable antimicrobial activity. Pharmacophore elucidation of the compounds was performed based on in silico ADMET evaluation of the tested compounds. Screening results of drug-likeness rules showed that all compounds follow the accepted rules, meet the criteria of drug-likeness and follow Lipinski’s rule of five. In addition, the toxicity results showed that all compounds are nonmutagenic and noncarcinogenic.
